# Generation of Novel Immunocompetent Mouse Cell Lines to Model Experimental Metastasis of High-Risk Neuroblastoma

**DOI:** 10.3390/cancers15194693

**Published:** 2023-09-23

**Authors:** Mayura R. Dhamdhere, Dan V. Spiegelman, Lisa Schneper, Amy K. Erbe, Paul M. Sondel, Vladimir S. Spiegelman

**Affiliations:** 1 Division of Pediatric Hematology and Oncology, Department of Pediatrics, The Pennsylvania State University College of Medicine, Hershey, PA 17033, USA; 2 Department of Human Oncology, School of Medicine and Public Health, University of Wisconsin, Madison, WI 53792, USA; d.spiegelman@wustl.edu (D.V.S.); pmsondel@humonc.wisc.edu (P.M.S.); 3 Department of Biochemistry and Molecular Biology, The Pennsylvania State University College of Medicine, Hershey, PA 17033, USA

**Keywords:** high-risk neuroblastoma metastasis, experimental metastasis model, HR-NB immunocompetent mouse model

## Abstract

**Simple Summary:**

Neuroblastoma (NB) is the most common extracranial solid pediatric cancer, with highly observed metastasis at diagnosis. Primary NBs generally have favorable outcomes with current therapies; however, high-risk, metastatic NBs are resistant and result in relapsed disease. Patients with metastatic NBs, thus, have a lower overall survival. Thus, to develop effective therapies for metastatic NBs, it is essential to better understand the biology of metastasis in NB. This requires the utilization of pre-clinical animal models, importantly, immunocompetent models that have an intact immune system and better reflect the disease physiology. However, currently, there are few good animal models available for studying HR-NB metastasis. This study aimed to generate efficient and appropriate mouse models to study HR-NB metastasis. Our newly developed and validated mouse cell lines are relevant and promising tools to study (high-risk) HR-NB metastasis and identify novel therapeutic targets for metastatic NBs; thus, they could benefit the NB research community.

**Abstract:**

NB, being a highly metastatic cancer, is one of the leading causes of cancer-related deaths in children. Increased disease recurrence and clinical resistance in patients with metastatic high-risk NBs (HR-NBs) result in poor outcomes and lower overall survival. However, the paucity of appropriate in vivo models for HR-NB metastasis has limited investigations into the underlying biology of HR-NB metastasis. This study was designed to address this limitation and develop suitable immunocompetent models for HR-NB metastasis. Here, we developed several highly metastatic immunocompetent murine HR-NB cell lines. Our newly developed cell lines show 100% efficiency in modeling experimental metastasis in C57BL6 mice and feature metastasis to the sites frequently observed in humans with HR-NB (liver and bone). In vivo validation demonstrated their specifically gained metastatic phenotype. The in vitro characterization of the cell lines showed increased cell invasion, acquired anchorage-independent growth ability, and resistance to MHC-I induction upon IFN-γ treatment. Furthermore, RNA-seq analysis of the newly developed cells identified a differentially regulated gene signature and an enrichment of processes consistent with their acquired metastatic phenotype, including extracellular matrix remodeling, angiogenesis, cell migration, and chemotaxis. The presented newly developed cell lines are, thus, suitable and promising tools for HR-NB metastasis and microenvironment studies in an immunocompetent system.

## 1. Introduction

Neuroblastoma (NB) is the most common extracranial solid cancer in children, and it accounts for around 15% of cancer-related deaths in pediatric patients [1,2,3,4]. Approximately half of all NB patients are classified as having high-risk (HR) metastatic disease [5]. Despite recent multimodal therapeutic regimens, including chemotherapy, surgical resection, radiation, autologous stem cell transplant, and GD2-immunotherapy, the 5-year survival in the HR group still remains <50% [1,2], with overall survival of only 20% for recurrent/relapsed NBs [5,6]. Moreover, HR-NB in general is a highly metastatic disease since the majority of children with HR-NB present with distant site metastases at diagnosis [2,3,4,7]. In addition, these patients with metastases in different organs have shown heterogeneous outcomes [7,8]. Thus, it becomes essential to understand the biology, microenvironment, and mechanisms underlying the metastasis process in HR-NB, which raises a need to expand the scope of existing pre-clinical models to study HR-NB metastasis.

Metastasis is a multistage event that is largely regulated via the microenvironment, consisting importantly of immune cells at the primary tumor location, as well as secondary sites. The utilization of immunocompetent pre-clinical models is, thus, essential to examine the progression of metastasis. Current pre-clinical HR-NB models include the xenograft implantation of human NB cells in immunocompromised mice, injecting immunocompetent murine cell lines (such as NXS2, Neuro-2A, and 9464D) in their respective syngeneic mouse models, and transgenic mice that develop spontaneous tumors [9,10,11,12,13]. Xenograft immunodeficient models are widely used to investigate small molecules; however, these are inappropriate for immunological studies or for assessing agents that depend on an intact immune system. The syngeneic line Neuro2A derived from the A/J mouse has been used for some immunological studies, although it has limitations due to A/J mice’s genetic background [9]. NXS2 cells (a hybrid generated by fusing the C1300 cell line from the A/J background and C57BL/6 mouse ganglia cells) [13] can be injected by IV to induce experimental metastases and can develop spontaneous metastasis. However, the development of spontaneous metastasis requires that the implanted tumor inoculum is given sufficient time for metastases to seed and grow, which normally requires the surgical excision of the more rapidly growing “primary tumor” prior to requiring a sacrifice to provide sufficient time for the detection of spontaneous metastases [13,14,15]. Also, unlike clinical HR-NBs, the NXS2 model is derived from a hybrid cell line, does not have MYCN amplification or overexpression, and has a high tumor mutational burden [16]. Thus, the NXS2 model does not resemble the clinical HR-NBs. The widely known TH-MYCN transgenic model overexpressing the MYCN oncogene was developed to reflect the MYCN-amplified HR-NB tumors [17]. This model has been reported to develop aggressive, spontaneous primary tumors that closely resemble human HR-NBs in terms of cytogenetic aberrations, gene expression profile, histology, and location, making it a relevant system for HR-NB studies [9,10,12]. The TH-MYCN transgenic mouse models can be studied for their development of spontaneous tumors (and maybe metastatic disease), but due to the heterogeneous timing of when tumors develop, these models are difficult to use when trying to test several groups of mice with identically sized tumors in randomized studies of various therapeutic interventions.

The 9464D NB cell line was derived from the spontaneous tumor of the TH-MYCN mouse and efficiently forms primary tumors in subcutaneous or orthotopic models [10]. This 9464D tumor model is MYCN-driven and can be implanted for growth in syngeneic immunocompetent C57Bl/6 mice. Unlike NXS2, the 9464D model has similar characteristics to human HR-NB, with a low tumor mutation burden, overexpression of MYCN, and aberrant expression of MHC-I, whereby only about 25% of 9464D are capable of MHC-I induction upon IFN-γ stimulation [9,18]. However, 9464D cells fail to form spontaneous metastases and have a very low penetrance of developing metastatic HR-NB, even via experimental metastasis. Thus, the current study was designed with an aim to overcome this limitation and generate efficient syngeneic cell lines to model experimental metastasis for HR-NB metastasis studies.

We developed several cell lines from 9464D-derived metastatic tumors (C57Bl/6 background) that model experimental metastasis in immunocompetent mice with 100% efficiency. Here, we present two highly metastatic cell lines (M1 and M2) derived from the liver metastasis of 9464D cells and several M1 variants derived from different metastatic tumors in different locations. Given sufficient time, these cell line models developed metastasis in all the IV-implanted animals. This study characterizes M1 and other newly developed 9464D variant cell lines, comparing them to the original 9464D. In vivo validation showed that the newly developed cell lines not only have a significantly higher metastatic potential but also exhibit organ-specific metastasis, particularly in the bone and liver, two prominently observed sites of metastases in HR-NB patients [8]. In vitro analysis showed that M1, M2, and M1 variants specifically acquired anchorage-independent growth and increased cell invasion ability. These cell lines are resistant to IFN-γ exposure, especially MHC-I upregulation, which is a characteristic of most HR-NBs, as shown for the primary 9464D cells [18]. Importantly, RNA-seq analysis revealed a differential gene expression signature of M1 cells, with enrichment of the pathways associated with cell survival, migration, angiogenesis, and extracellular matrix remodeling, that can potentially contribute to the acquired metastatic potential of these cells.

## 2. Materials and Methods

### 2.1. Cell Lines

Mouse neuroblastoma cell line 9464D was cultured in Dulbecco’s modified Eagle’s medium (DMEM; VWR International, Radnor, PA, USA), supplemented with 10% *v*/*v* fetal bovine serum (FBS, Gibco by Life Technologies, Carlsbad, CA, USA) and 100 units/mL of penicillin and streptomycin (Corning, Corning, NY, USA). Stable 9464D-luciferase reporter cells were generated as previously mentioned, using pCDH-EF1-Luc-p2a-tdTomato (RRID: Addgene_72486) lentiviral transduction, followed by tdTomato fluorophore sorting. The cells were then verified for luciferase activity using the Promega luciferase assay system (Promega, Madison, WI, USA, E1500).

#### 2.1.1. M1 and M2 Cell Line Generation

Initially, in order to determine a workable cell number for experimental metastasis for our previous study, 9464D (Luc-tdT) cells were IV-injected at different cell titers (0.25, 0.5, and 1 million/mouse) in C57Bl/6J mice [19] (Jackson Laboratory, Bar Harbor, ME, USA, RRID: IMSR_JAX:000664). Only 2 out of 30 mice injected with 9464D cells developed metastasis, and the liver tumors from these two mice were isolated and processed as previously described [19]. Briefly, isolated tumors were washed with sterile Dulbecco’s phosphate buffered saline (DPBS), minced, and digested sequentially with collagenase/dispase/trypsin. Resuspended cell pellets were passed through 18G, 23G, and 27G needles to obtain single-cell suspensions. Different cell populations were separated out using the pre-plating technique, and further homogeneous purified NB cells were obtained via a differential trypsinization and replating procedure. These cells were then sorted for tdTomato expression (tdt), validated using MYCN expression, and named M1 and M2. The M1 line was used in our recently published study, which demonstrated the importance of IGF2BP1 in NB metastasis [19].

#### 2.1.2. Generation of M1-Derivative Lines

Likewise, to obtain additional organ-specific metastatic cell lines, a higher titer of M1 cells (2 million/mouse) was IV-injected into C57Bl/6J mice. These mice were monitored for the growth of experimental metastasis via in vivo bioluminescence imaging. Tumors from different locations, relevant to the prominently observed human primary and metastatic HR-NB sites, were isolated and processed as mentioned above. The obtained homogeneous HR-NB cell lines were validated using MYCN expression and named M1-1 (derived from lymph nodes), M1-2 (bone and the hindlimb), M1-3 (the stomach/intestine), M1-4 (the thoracic region (ganglia)), M1-5 (bone and craniofacial), M1-6 (the adrenal gland), and M1-7 (the lumbar vertebrae/ganglia). All the lines were cultured using the same DMEM medium as above. All the above parental and generated cell lines were regularly tested for mycoplasma contamination (MycoAlert plus mycoplasma detection kit, Lonza Bioscience, Morristown, NJ, USA) and were cultured for less than a month for the experiments.

### 2.2. In Vivo Characterization

Six-to-eight-week-old C57Bl/6J mice were used, and the animals were randomly assigned to each group (*n* ≥ 4 animals/group) for all experiments. No blinding was performed. For primary tumor growth characterization, 0.5 × 10^6^ 9464D, M1, M2, or M1-variant cells were injected subcutaneously, and the mice were monitored for tumor growth and survival. Tumor sizes (diameter, in mm) were measured twice a week until they reached the experimental endpoint size of ≥30 mm.

To validate their efficiency for experimental metastasis, 1 × 10^6^ 9464D, M1, M2, or M1-variant cells were injected intravenously (tail vein), followed by monitoring these mice for metastasis growth using an in vivo bioluminescence imaging system. The mice were anesthetized using 2.5% isoflurane (IsoSol Isoflurane, USP, VEDCO, St Joseph, MO, USA) and injected with 0.15 mg/g body weight of D-Luciferin (D-Luciferin Potassium salt # LUCK-100, Goldbio, St Louis MO, USA) dissolved in PBS 10 min prior to imaging. The mice were imaged once or twice a week. The animals were followed for survival analysis. Humane euthanasia/death was the experimental endpoint for these metastasis experiments. All in vivo mouse studies were approved by the Institutional Animal Care and Use Committee (IACUC) at the Pennsylvania State University College of Medicine.

### 2.3. Cell-Based In Vitro Functional Assays

#### 2.3.1. IncuCyte Cell Proliferation and Migration Assays

The 9464D, M1, M2, and M1 variants’ M1-2, M1-5, and M1-6 cells were seeded at 5000 cells/well in 96 well plates for proliferation assays. Live-cell phase contrast images were obtained using a 10× objective lens (three images per well), and cell confluence was analyzed using IncuCyte Live Cell Analysis (v2019B) software. For migration assays, 40,000 cells/well (100 μL/well) were seeded in IncuCyte ImageLock 96-well plates (Sartorius 4379, Niedersachsen, Germany), and after 12–14 h, scratches were made using the IncuCyte 96-well Wound Maker Tool (Sartorius 4563), as per the manufacturer’s protocol. Images were obtained every 2 h for 72 h, and relative wound density was quantified using the Incucyte Cell Migration Analysis software (Sartorius 9600-0012).

#### 2.3.2. Invasion Assay

Invasion assays were performed using the Corning Biocoat Matrigel invasion chambers (Corning 354480) and control inserts (Corning 354578), as per the manufacturer’s protocol. Briefly, 25,000 cells in 500 μL of serum-free media were seeded in the inserts, and complete media (+10% FBS) was added to the wells of the companion plate as a chemoattractant. After 48 h, the inserts were fixed and permeabilized by subjecting them to 4% (*v*/*v*) formaldehyde (in PBS), followed by 100% methanol. The inserts were then stained with 0.25% crystal violet (in 20% methanol), rinsed, dried, and analyzed for invaded cell numbers under a 20× phase contrast microscope. Images from 7–8 random areas were acquired using the Biorad ZOE Fluorescent cell Imager. The area of invaded and migrated cells from the Matrigel and control inserts, respectively, was quantified using the ImageJ software (https://imagej.nih.gov/ij/index.html (accessed on 22 January 2021)). Cell invasion data is expressed as % invasion through Matrigel matrix inserts relative to migration through the control inserts (formulae: % invasion = area of invaded cells through Matrigel inserts/area of migrated cells through control inserts × 100).

#### 2.3.3. Colony Formation Assay

Single cells were seeded at a density of 1000 cells/well in 6 well plates and allowed to grow until the appearance of prominent colonies. On day 6, the wells were washed twice with PBS, and the colonies were fixed using 4% formaldehyde, followed by 100% methanol for 5 min each. The colonies were then stained with 0.25% crystal violet (in 20% methanol) for 20 min at room temperature (RT) and rinsed with tap water. The plates were dried on a bench top for 2 days, and images were acquired using the ChemiDoc MP imaging system (Bio-Rad, Hercules, CA, USA) and analyzed using ImageJ (https://imagej.nih.gov/ij/index.html (accessed on 22 January 2021)).

#### 2.3.4. Soft Agar Assay

Soft agar colony formation was used to examine the anchorage-independent growth ability of cells, following an established protocol [20] with minor modifications. Briefly, for the base layer, 1% warm agar was mixed with prewarmed (at 42 °C) DMEM (with 10% FBS, 1% penicillin and streptomycin) at a 1:2 dilution, and 1.5 mL of this mixture was layered per well in the 6 well plates. The plates were allowed to solidify for 15 min at RT. The cells were resuspended to attain a concentration of 1500 cells/750 µL of media (prewarmed, 42 °C) and mixed with 750 µL of 0.6% of warm (42 °C) agar solution. This mixture was then layered (1.5 mL/well) above the solidified base agar layer in 6 well plates and allowed to solidify in a humidified incubator at 37 °C. On top of the agar layers, 500 µL of complete DMEM (+10%FBS and 1%P/S) was maintained as a feeder medium, and the plates were monitored regularly until the appearance of colonies, which in this case occurred at around 9–10 days. Ten days post cell seeding, the liquid feeder medium was removed, and the colonies were stained with 0.05% crystal violet (in 20% methanol) for an hour in a 37 °C incubator. Excess stain was removed via washing several times with water. Six to eight images per well were acquired from different regions, and the images were analyzed using ImageJ to quantify the colony number and size (Feret’s diameter).

### 2.4. Western Blot

Western blotting was performed as previously described [19]. Briefly, 60 µg of total protein was separated on 10% polyacrylamide/SDS gel and transferred to PVDF membranes, and the membranes were blocked with 5% (*w*/*v*) bovine serum albumin (BSA) in tris buffered saline (TBS) (+0.1% Tween-20). The primary antibodies included N-Myc (Cell Signaling Technology Cat # 84406, RRID: AB_2800038, Danvers, MA, USA), β-Actin (Cell Signaling Technology Cat # 4970, RRID: AB_2223172), and horseradish peroxidase-conjugated secondary antibody: anti-rabbit IgG (Cell Signaling Technology Cat# 7074, RRID: AB_2099233).

### 2.5. Flow Cytometry and Surface Staining

For MHC-I and PD-L1 expression analysis, 2.5 × 10^5^ cells were plated in 6 well plates in 2 mL of DMEM media (+10% heat-inactivated FBS (Gibco), 2 mmol/L of *L*-Glutamine (Corning), 100 U/mL of penicillin, and 100 μg/mL of streptomycin (Corning)). After 24 h, the cells were treated with 1000 U/mL of IFN-γ (200U, BioLegend, San Diego, CA, USA, Cat# 575308). Forty-eight h post-treatment, the cells with or without IFN-γ were harvested, washed with a FACs buffer (PBS + 2% FBS), and centrifuged at 300× *g*. The pelleted cells were resuspended in around 50 µL of FACs buffer and stained with APC anti-mouse H-2Kb/H-2Db (BioLegend Cat # 114614, RRID: AB_2750194) antibody for 30 min at RT in the dark. Following a washing step (300× *g* for 5 min), a drop of DAPI (BioLegend, Cat # 422801) was added to the cell suspensions, and data were acquired using a ThermoFisher Attune NxT flow cytometer. The data were analyzed using FlowJo v.10.8.1.

For GD2 staining, the cells were harvested via trypsinization and counted. Then, 1 × 10^6^ cells were resuspended in 200 µL of PBS and washed once via centrifuging (1500 rpm, 5 min). The pelleted cells were resuspended in 150 µL of FACs buffer (PBS with 2% FBS + 0.1% sodium azide), and centrifuged as above. The cell pellets were resuspended in 50 µL of FACs buffer and stained with FITC anti-human Ganglioside GD2 (BioLegend Cat # 357314, RRID: AB_2800977) for 30 min on ice in the dark. The cells were then washed (1500 rpm, 5 min) twice with 150 µL of FACs buffer, fixed with 200 µL of 2% paraformaldehyde for 20 min on ice, and, at the final step, resuspended in 300 µL of FACs buffer. As a positive control, 9464D-GD2 cells, generated via GD2/GD3 synthase (pCDH1-CMV-St8sia1-2A-B4galnt1-SV40-Hygro [21]) lentiviral transduction, were used. Data were acquired via the BD LSRFortessa (BD Biosciences, San Jose, CA, USA.) flow cytometer and analyzed using the FlowJo v.10.8.1 software.

### 2.6. RNA-Seq and Differential Gene Expression Analysis

#### 2.6.1. Library Preparation and Sequencing of mRNA (Illumina Kit)

Three biological replicates of 9464D and M1 cells were collected, and total RNA isolation was done using a TRIzol reagent (Invitrogen, Carlsbad, CA, USA), as per the manufacturer’s protocol, before being subjected to RNA-seq. RNA-seq libraries were prepared in the Penn State College of Medicine Genome Sciences core (RRID: SCR_021123) using the Illumina Stranded mRNA Prep Ligation kit (Illumina, San Diego, CA, USA), as per the manufacturer’s instructions. Briefly, polyA RNA was purified from 200 ng of total RNA using oligo (dT) beads. The extracted mRNA fraction was subjected to fragmentation, reverse transcription, end repair, 3′– end adenylation, and adaptor ligation, followed by PCR amplification and magnetic bead purification (Omega Bio-Tek, Norcross, GA, USA). The unique dual index sequences (IDT^®^ for Illumina RNA UD Indexes Set C, Ligation, Illumina) were incorporated into the adaptors for multiplexed high-throughput sequencing. The final product was assessed for its size distribution and concentration using a BioAnalyzer High Sensitivity DNA Kit (Agilent Technologies, Santa Clara, CA, USA). The libraries were pooled and sequenced via Illumina NovaSeq 6000 (Illumina) to get, on average, 25 million paired-end 50 bp reads, according to the manufacturer’s instructions.

#### 2.6.2. Differential Gene Expression and Pathway Analysis

De-multiplexed and adapter-trimmed sequencing reads were generated using Illumina bcl2fastq (released version 2.18.0.12), allowing no mismatches in the index read. The reads were aligned with the *Mus musculus* GRCm39 reference genome using STAR v.2.7.3a with the quantMode Gene Counts option to generate read counts per gene [22]. Differential expression analysis was performed in R (v. 4.2.1) (R Core Team) using edgeR (v. 3.16) [23]. Briefly, read counts were modeled for each gene in each sample using the negative binomial model along with the generalized linear model, and differentially expressed genes were determined using the *t*-tests relative to a threshold (TREAT) method described by McCarthy and Smyth with a significant absolute log2 fold change = 1 [24]. Differentially expressed genes were visualized using the ggplot2 (v 3.4.2) (Wickham H, 2016) and NMF (v. 0.26) [25] R packages. Significance was defined for those with q-values < 0.05 calculated via the Benjamini–Hochberg method to control the false discovery rate (FDR). Initial pathway analysis was performed with the ClusterProfiler (v. 4.6.2) R package [26,27], and terms were summarized using REVIGO [28]. Significant differentially expressed genes were further divided into two groups—upregulated and downregulated—and were analyzed using Metascape (http://metascape.org (accessed on 17 February 2023)) [29] to identify the GO enriched terms—biological processes and canonical pathways.

### 2.7. Statistical Analysis

GraphPad Prism 9 was used for statistical analysis. One-way ANOVA was used to compare multiple groups, and a non-linear regression model was used to compare tumor growth rates. The data are presented as means ± SEMs. Survival curves were generated using Kaplan–Meier survival analysis. Statistical significance is indicated on the graphs, as well as in the figure legends (significance level: α = 0.05).

## 3. Results

### 3.1. Generation of M2- and M1-Derived Cell Lines

**Figure 1:**
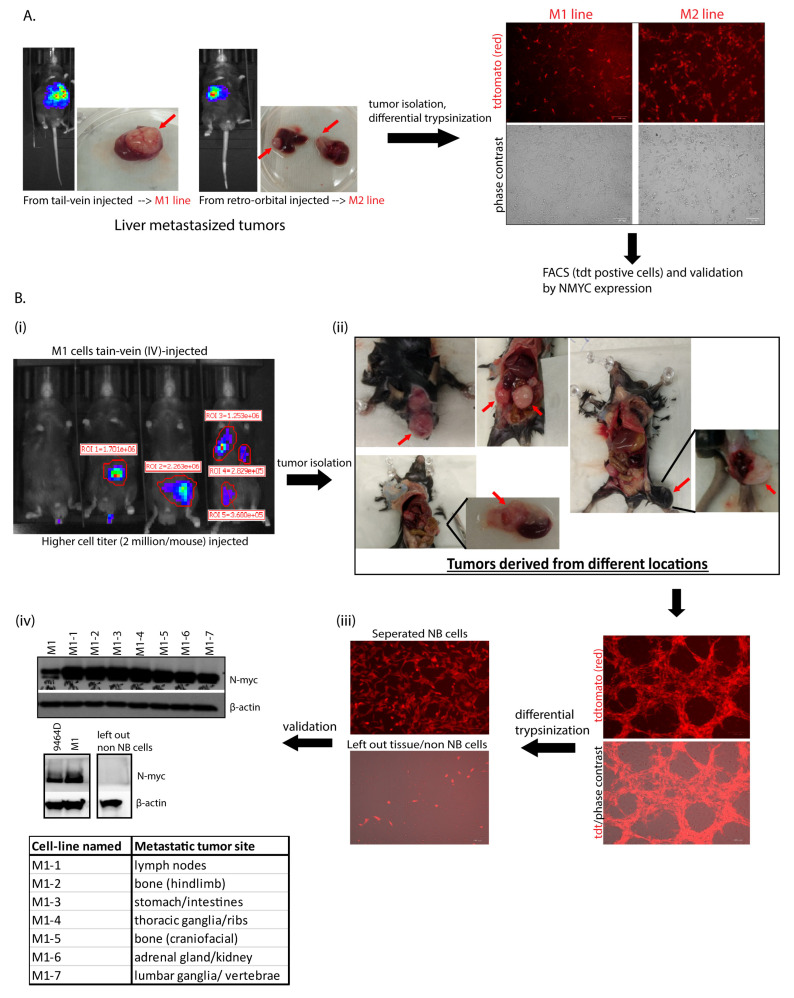
Generation of cell lines. (**A**) Establishment of M1 and M2 lines from isolated liver tumors (indicated with red arrows); two out of thirty mice with parental-9464D cell-injected (IV) via the tail vein and retro-orbital route developed metastasis, and their tumors were isolated and processed for cell line generation. tdTomato expression in the cells confirms their derivation from the original tdT^+^ 9464D cells injected by IV. (**B**) Generation of M1 variants and validation using N-Myc expression. (Five mice were injected with a higher cell titer, 2 million M1 cells/mouse by IV.) Tumors from different locations (the lymph nodes, bone (craniofacial and the hindlimb), the stomach/intestines, the kidney/adrenal gland, the thoracic ganglia/ribs, and the lumbar ganglia/vertebrae) were isolated and processed via the differential trypsinization method to separate out pure NB tumor cells from other non-NB/tissue cells (tumors indicated with red arrows). Western blot was used for N-Myc expression in all the M1-derived lines and left out non-NB/tissue cells. β-actin was used as a load control. Original western blots are presented in File S1.

Due to the inefficiency of the original parent 9464D cells to model experimental metastasis, we generated several highly metastatic transplantable cell lines. Out of thirty mice intravenously (IV) injected with 9464D cells, only two developed metastatic tumors. Liver tumors from both these mice were isolated and processed to generate established lines—named M1 and M2, respectively (Figure 1A). Their expression of tdTomato confirmed that they were derived from the original tdTomato^+^ 9464D cells injected by IV. In addition, the M1 and M2 cells exhibited the overexpression of N-Myc, similar to 9464D (Supplementary Figure S1), validating their 9464D-HR-NB origin. In order to generate additional organ-specific lines, a higher titer of M1 cells (2 million cells/mouse) was injected by IV into C57BL/6J mice (Figure 1(Bi)). Metastatic tumors were found in various locations relevant to observed primary/metastatic sites in HR-NBs patients [2,3,4,7,8]. These were isolated and processed to establish additional variant cell lines—named M1-1 to M1-7. The locations where these metastases were found included the adrenal gland, axillary lymph nodes [30], thoracic ganglia, lumbar vertebrae/ganglia, stomach, and two bone sites—craniofacial and the hindlimb (Figure 1(Bii)). Purified HR-NB cells obtained via differential trypsinization were validated using MYCN expression—a marker for 9464D-derived HR-NB cells. The derived NB cells (but not non-NB/tissue cells) expressed tdTomato and N-Myc, thus confirming their derivation from the tumors that developed upon injecting the M1 cells (Figure 1(Biii,Biv)).

### 3.2. Primary Tumor Growth and Survival Kinetics Using Subcutaneous Model

**Figure 2:**
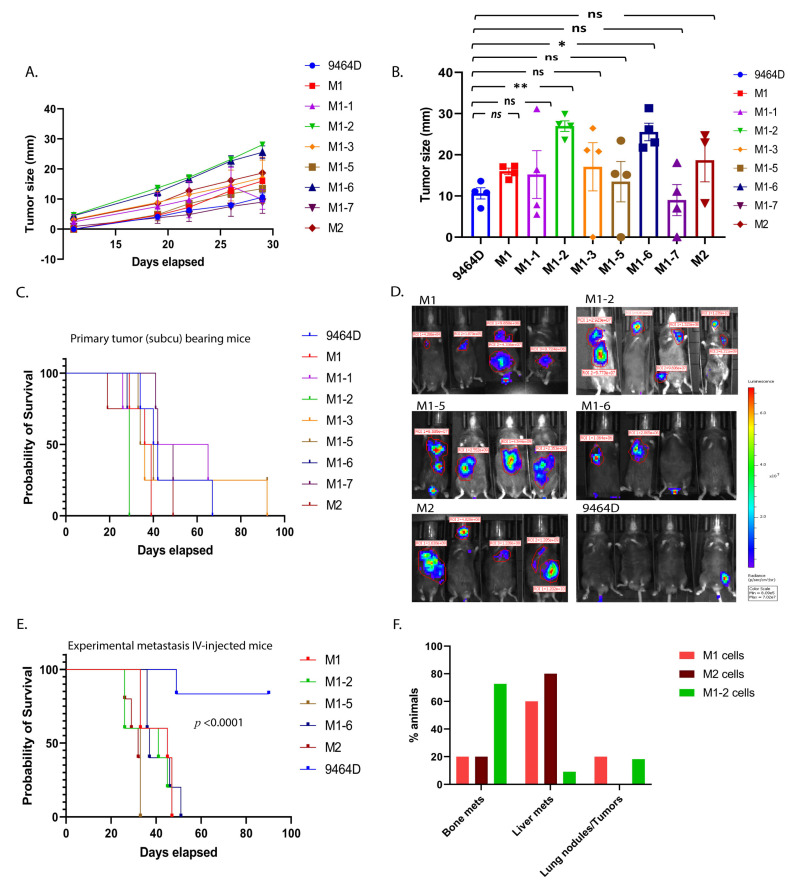
In vivo characterization of M1, M2, and M1-derived novel transplantable mouse HR-NB cell lines. (**A**) Efficiency of cell lines in forming primary tumors. C57Bl6 mice were injected subcutaneously (0.5 million cells/mouse, n = 4 animals per group), and their tumor sizes were measured twice a week until they were euthanized due to their tumor burden (statistical test—non-linear regression model, F-test, 9464D vs. M1-2, *p*-value <0.0001, and 9464D vs. M1-6, *p*-value <0.0001, 9464D vs. M1, M1-1, M1-3, M1-5, and M-7, *p*-value—ns (0.692), 9464D vs. M2 *p*-value—ns (0.199)). (**B**) Tumor sizes at day 30 post-cell-injections (statistical test—Welch’s ANOVA test, mean ± SEM, p-values: 9464D vs. M1—ns (0.232), 9464D vs. M1-1—ns (0.999), 9464D vs. M1-2— ** (0.002), 9464D vs. M1-3—ns (0.989), 9464D vs. M1-5—ns (0.999), 9464D vs. M1-6— * (0.030), 9464D vs. M1-7—ns (0.999) and 9464D vs. M2—ns (0.899)). (**C**) Survival affected by primary tumors—Kaplan–Meier analysis, log-rank (Mantel–Cox) test, 9464D vs. M1-2: *p*-value—0.010, 9464D vs. M1-6: *p*-value—0.010, 9464D vs. M1, M1-1, M1-3, M1-5, M1-7, and M2: *p*-value—ns (0.4380). (**D**) Ability of cells to form metastasis using an experimental metastasis approach. Metastasis monitored via bioluminescence in vivo imaging (in vivo bioluminescence image—week 4 post-cell-injection, 1 million cells/mouse IV-injected (the tail vein), n = 5 animals/group). Although some of the mice lost luciferase expression (luminescence), all of them developed metastases. (**E**) Mortality due to metastasis for the mice shown in (**D**)—Kaplan–Meier analysis, log-rank (Mantel–Cox) test, *p*-value <0.0001. (**F**) Tumor sites, fraction of total mice IV-injected with NB cells, with subsequent metastasis in the indicated location, represented as percentages (M1 cells: bone—20%, liver—60%, and lung—20%; M2 cells: bone—20%, liver—80%, and lung—0%; and M1-2 cells: bone—73%, liver—9%, and lung—18%).

Initially, the newly generated cell lines were examined for their ability to form primary tumors, comparing them to 9464D cells. For M1, M2, and all M1-derived subcutaneous models except M1-4, tumor growth was observed in all mice inoculated subcutaneously. Specifically, M1-2 and M1-6 exhibited significantly faster tumor growth over the period of monitoring, with the appearance of palpable tumors at week 1 post-cell-injections (Figure 2A). Irrespective of the differential growth rates, all newly developed cell lines except M1-7 (though not significantly lower), showed tumor sizes higher than or comparable to 9464D at the experimental endpoint of day 30 post-cell-inoculation (Figure 2B). Consequently, the mice bearing M1, M1-1, M1-3, M1-5, or M2 tumors showed slightly lower (though not statistically significantly) or similar survival as that of 9464D-inoculated mice (Figure 2C), while the M1-2- and M1-6-tumor-bearing mice had significantly lower survival due to their primary tumor burden.

### 3.3. M1 and M2 Cells and the M1 Derivatives M1-2 and M1-5 Show Increased Metastatic Ability

Based on their derived locations (bone, the kidney/adrenal gland, and the liver) and relevance to the prominently observed metastatic sites in HR-NB patients, we further chose M1-2, M1-5, M1-6, and M2, along with M1, to examine their potential to generate metastases after IV injections. As previously demonstrated [19], the M1 cells consistently showed a higher metastatic ability, with the liver being the preferred site of metastasis. Similarly, M1-2, M1-5, M1-6, and M2 also had a higher metastatic phenotype (100% tumor penetrance) compared to 9464D (Figure 2D), and thus significantly lower mouse survival than 9464D (Figure 2E). Even though some of the mice lost luminescence (luciferase expression) over time and, thus, did not emit a luminescence signal, all the IV-injected mice did develop metastases. Importantly, like M1, the M1-2 and M2 cells exhibited organotropism, with M2 specifically developing mostly liver metastasis and M1-2 showing a preference for metastasizing to bones (Figure 2F). Taken together, the in vivo characterization confirmed that the novel M1, M2, and M1-derivative cell lines were more metastatic in these experiments than the parent 9464D cells and featured a prominent metastatic site preference consistent with human HR-NB metastases, thus making them efficient model systems for studying HR-NB metastasis using the IV-injected experimental metastasis approach.

### 3.4. In Vitro/in-Culture Characterization of New Cell Lines

The 9464D cell line is derived from the spontaneous tumor of the TH-MYCN mice and, thus, expresses high levels of MYCN [9], a signature of MYCN-amplified HR-NBs. Initially, to ascertain that established cell lines were of HR-NB origin and to determine whether any alterations to the MYCN transgene expression were present, we examined the expression of MYCN in these cells. M1, M2, and all the M1-derived lines expressed substantial levels of MYCN, similar to 9464D cells (Figure 1(Biv)), Supplementary Figure S1).

**Figure 3:**
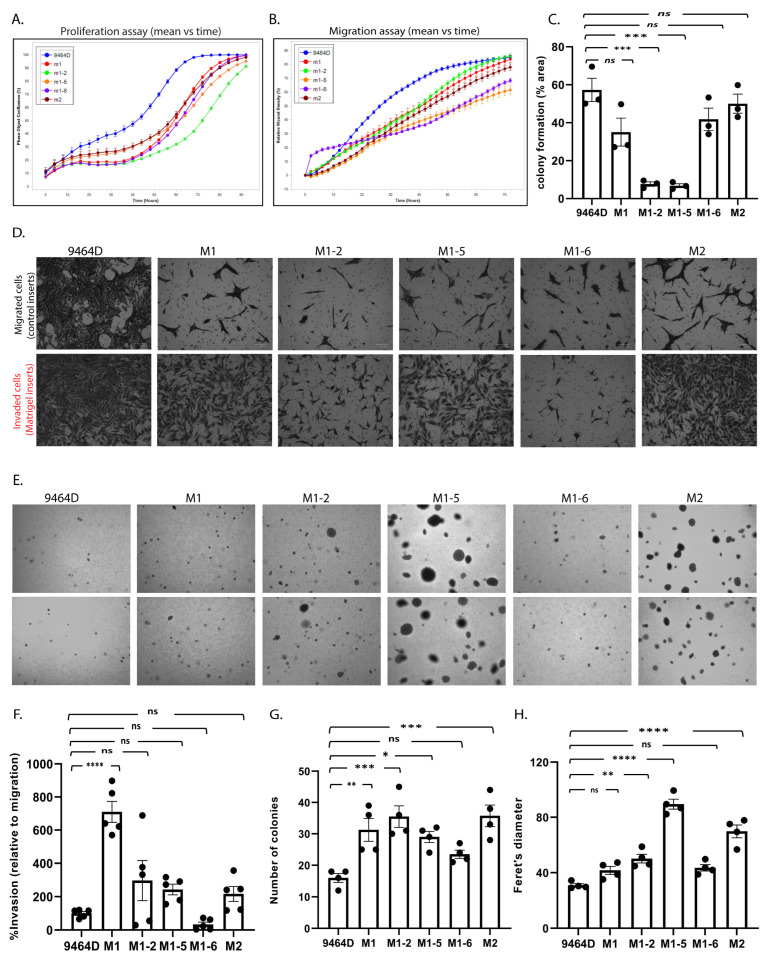
In vitro characterization of the cell line models. (**A**) Cell proliferation and (**B**) cell migration using the IncuCyte live-cell analysis system. (**C**) Colony formation assay, statistical analysis—one-way ANOVA, mean ± SEM, p-values: 9464D vs. M1—ns (0.076), 9464D vs. M1-2—*** (0.0002), 9464D vs. M1-5—*** (0.0002), 9464D vs. M1-6—ns (0.324), and 9464D vs. M2—ns (0.905). (**D**) Invasion assay using Biocoat Matrigel invasion inserts and control inserts. Data depict % invasion relative to migration. (**E**) Anchorage-independent (soft agar) assay. (**F**) Quantification of invasion assay. (**D**) Statistical analysis—one-way ANOVA, mean ± SEM, p-values: 9464D vs. M1—**** (< 0.0001), 9464D vs. M1-2—ns (0.2336), 9464D vs. M1-5—ns (0.5618), 9464D vs. M1-6—ns (0.9679), and 9464D vs. M2—ns (0.7475). (**G**,**H**) The quantification of the anchorage-independent assay (**E**), where (**G)** is the number of colonies in the soft agar assay, statistical analysis—one-way ANOVA, mean ± SEM, p-values: 9464D vs. M1—** (0.009), 9464D vs. M1-2—*** (0.0009), 9464D vs. M1-5—* (0.0319), 9464D vs. M1-6—ns (0.3991), and 9464D vs. M2—*** (0.0008) and (**H**) is the Feret’s diameter (colony size), statistical analysis—one-way ANOVA, mean ± SEM, p-values: 9464D vs. M1—ns (0.204), 9464D vs. M1-2—** (0.0049), 9464D vs. M1-5—**** (< 0.0001), 9464D vs. M1-6—ns (0.0989), and 9464D vs. M2—**** (< 0.0001). (Each assay was repeated in n ≥ 3 independent experiments.)

In addition, we evaluated the cellular phenotypes of these cells in culture, comparing them to 9464D cells. Cell proliferation, migration, invasion, colony formation, and anchorage-independent growth are the standard functional assays to assess the tumorigenic behavior of cancer cells. M1, M2, and M1-derived cells were examined for these in vitro cellular phenotypes. Both M1 and M2 cells showed reduced cell proliferation and migration compared to 9464D. Similarly, all the M1 derivatives exhibited a lower in vitro proliferation and migration rate relative to 9464D cells (Figure 3A,B and Supplementary Figure S2A,B). While M1-2 and M1-5 showed significantly lower clonogenicity compared to 9464D, M1, M1-6, and M2 retained this colony formation ability, similar to 9464D (Figure 3C, Supplementary Figure S2C). Interestingly, M1 cells showed a significantly higher invasion rate relative to 9464D. M2, M1-2, and M1-5 also showed increased cell invasion compared to 9464D (though not significantly higher) (Figure 3D,F). Moreover, all the newly developed lines formed a greater number of colonies and larger-sized colonies on soft agar plates than 9464D. Specifically, M1-5 and M2 formed the largest-sized colonies with Feret’s diameters of 89.5 and 69.8, respectively (Figure 3E,G,H). These data, thus, suggest that the M1, M2, and M1-derived cells specifically acquired an anchorage-independent growth and invasive property that potentially contributed to their highly aggressive, metastatic behavior in vivo. Despite being derived from M1-originated tumors, the M1 derivatives showed heterogeneity in the in-culture phenotypes, reflecting their differential selection in another in vivo passage at different metastatic sites from where they were isolated.

### 3.5. M1 Cells and Their Derived Variants Have Low Expression of GD2 and Are Resistant to MHC-I Induction via IFN-γ

**Figure 4:**
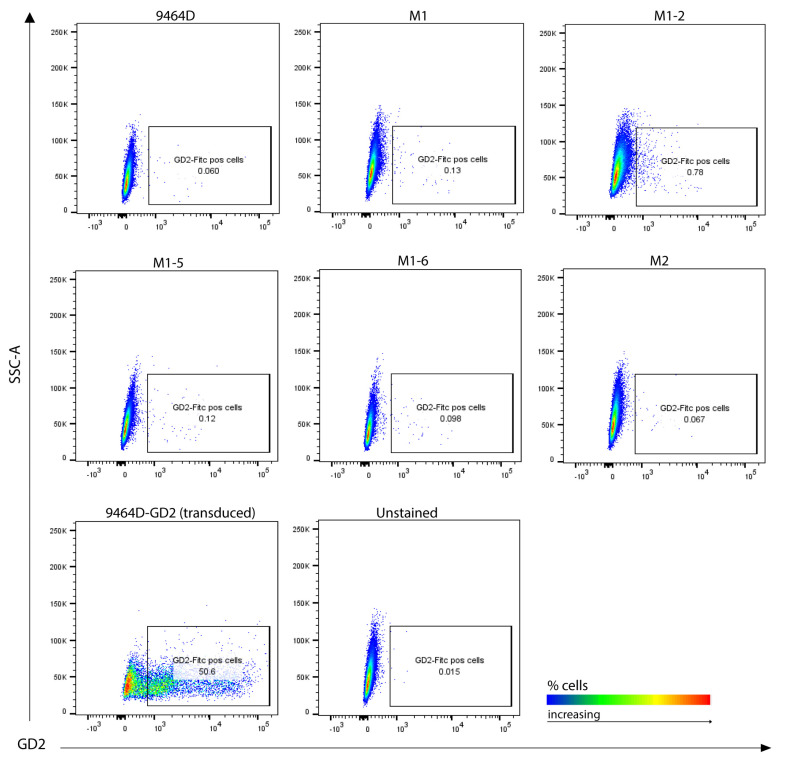
GD2 surface expression analysis. Cultured cells stained for flow cytometry, representative plots showing GD2 positive cells. GD2/GD3 synthase-transduced 9464D cells (9464D-GD2) were used as a positive control. Unstained cells were used as a background control.

**Figure 5:**
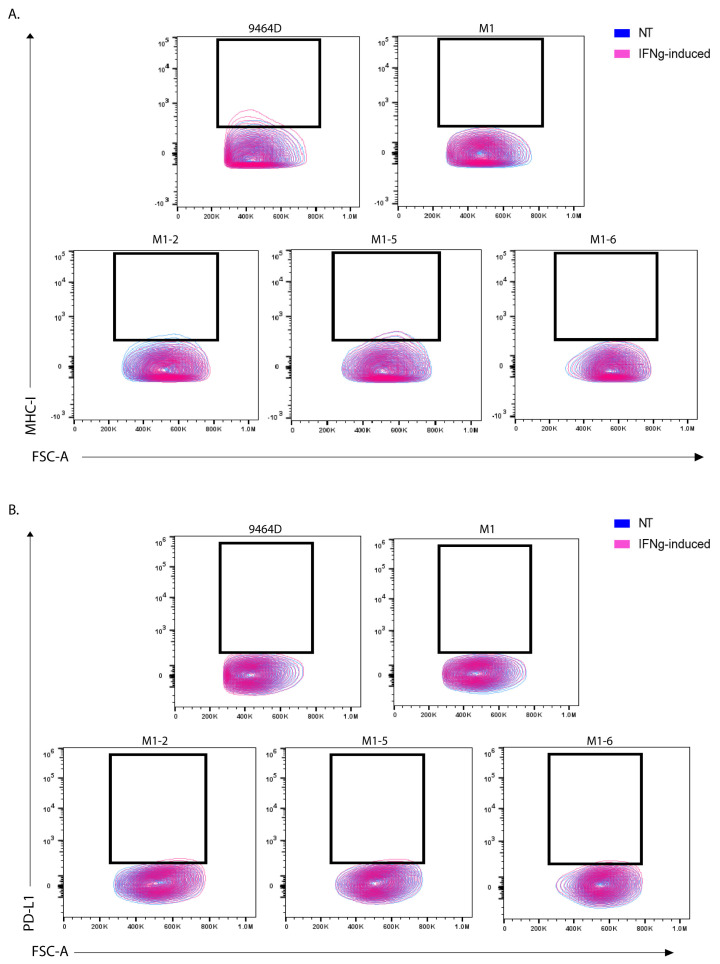
MHC-I and PD-L1 marker expression in M1 and M1-variant cells. Flow analysis for (**A**) MHC-I expression and (**B**) PD-L1 expression in non-treated (NT) vs. IFN-γ treated (IFNg) cells. Unstained cells from each cell line were used as a background control.

High GD2 expression is a characteristic of human NBs and is a potent target for current clinical immunotherapies for NB. Nearly all human HR-NB cell lines have high GD2 expression; however, most of the murine HR-NB cell lines, including 9464D, express very low levels of GD2. Furthermore, previous studies have shown the dynamic nature of GD2 expression on 9464D cells [9,11]. To obtain high levels of GD2, these cells need to be transfected with both GD2 and GD3 synthase, which (9464D-GD2) we used here as a positive control for GD2 staining. We assessed the newly developed cell lines for GD2 expression to investigate whether they exhibited altered GD2 expression. M1, the M1 variants, and M2 all showed similar, virtually absent levels of GD2 expression, comparable to the level of the original 9464D (Figure 4). In addition, we evaluated these cells for the characteristic immune markers (MHC-I and PD-L1), comparing them to the 9464D cells. IFN-γ is known to help induce anti-tumor immunogenicity by upregulating the expression of MHC-I and II on some cancer cells in multiple tumor types. In accordance with previous studies [9,18], 9464D cells showed low levels of MHC-I surface expression, and a fraction of these cells showed MHC-I upregulation upon IFN-γ induction [18]. However, the M1 cells expressed substantially lower levels of MHC-I relative to the 9464D cells and, importantly, failed to upregulate them upon IFN-γ induction (Figure 5A, Supplementary Figure S3), indicative of a less immunogenic phenotype. The M1 variants also showed lower MHC-I and failed to upregulate its expression upon IFN-γ stimulation. PD-L1 is expressed in a very low number of 9464D cells and the newly developed M1 and M1-variant cells (Figure 5B). Overall, the immune characterization revealing lower MHC-I expression in M1 and the M1 variants, and their insensitivity to phenotype changes in response to IFN-γ, suggests an “immunologically cold” phenotype for these M1 cells and their variants, as is often characteristic of human HR-NBs.

### 3.6. RNA-Seq Analysis Reveals a Differential Gene Expression Profile of M1 Cells

**Figure 6:**
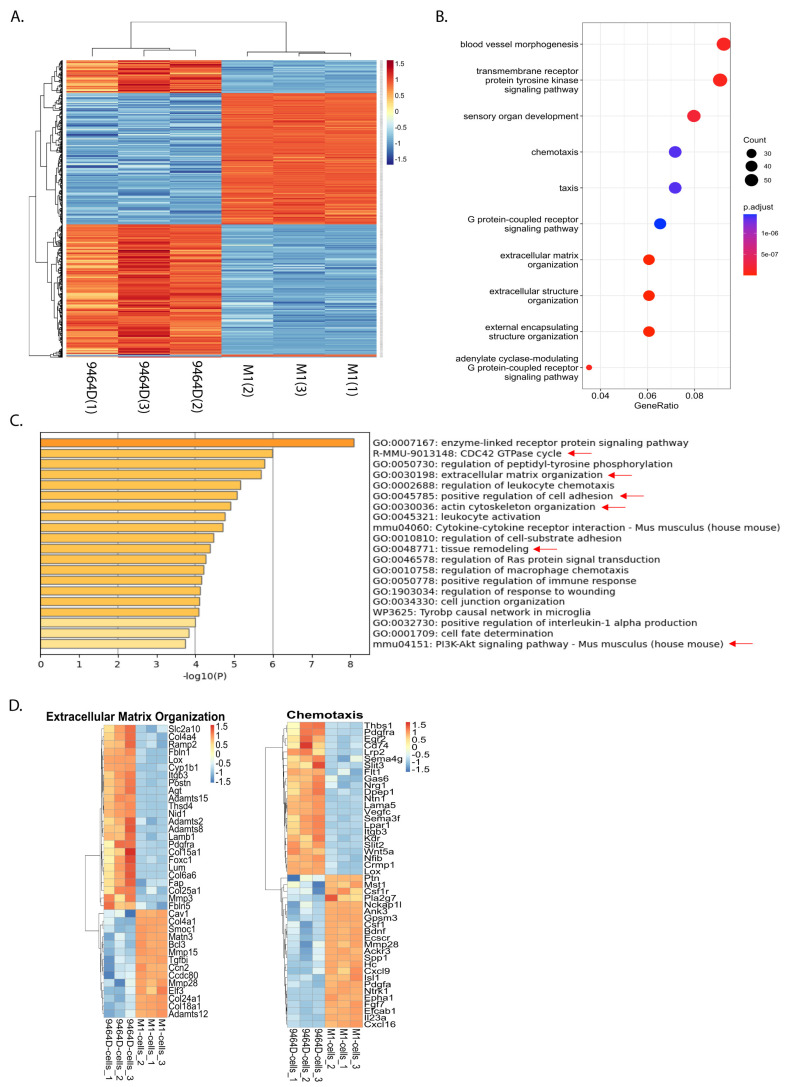
M1 cells show a differentially regulated gene expression profile: 9464D or M1 cells were subjected to RNA-seq and differentially expressed gene signatures were examined. (**A**) Heatmap showing clusters of differentially regulated genes in M1 cells relative to 9464D cells (*n* = 3 replicates, an FDR of 0.05 set to identify the top hits). (**B**) Dot plot depicting the top significantly altered pathways. (**C**) Metascape Gene ontology analysis showing the enriched biological processes associated with upregulated genes in M1 cells (metastasis relevant processes indicated with red arrows). (**D**) Heatmaps depicting differentially regulated genes associated with extracellular matrix organization and chemotaxis.

To determine the potential mechanisms responsible for the acquired metastatic ability, we compared transcriptomes of M1 and parental 9464D cells utilizing RNA-seq analysis. We found a substantial cluster of significantly up- and downregulated genes in M1, relative to the 9464D cells. A stringent FDR of 0.05 (5%) was set to identify the significant hits. These top-most differentially regulated (686) genes consisted of 308 upregulated and 378 downregulated genes (Figure 6A). Furthermore, gene ontology (GO) annotations revealed that differentially expressed genes were associated with blood vessel formation, extracellular matrix remodeling, cell migration, chemotaxis, cell–cell adhesion, and immune cell regulation (Figure 6B–D, Supplementary Figure S4). Importantly, the pathways specifically associated with upregulated genes consisted of extracellular matrix organization, actin cytoskeleton organization, blood vessel development and angiogenesis, the positive regulation of cell motility, migration and chemotaxis, cell adhesion, the CDC42 GTPase cycle, and the PI3K-Akt pathway (Figure 6C, Supplementary Tables S1 and S2).

In contrast, the downregulated genes were linked with biological processes, such as the positive regulation of apoptosis, the negative regulation of cell migration, negative chemotaxis, and the negative regulation of blood vessel endothelial cell migration and angiogenesis (Supplementary Table S2). Overall, this differential gene signature sheds light on the potential mechanisms underlying the acquired metastatic potential of this M1 model and, presumably, the M1 variants.

## 4. Discussion

In this report, we present novel, highly metastatic HR-NB syngeneic mouse models. These clinically relevant murine HR-NB models feature metastasis to the organs that are characteristic of human HR-NB metastasis. Due to the lack of a spontaneous metastasis model in NB, an experimental metastasis approach is the next best available strategy for studying metastasis in NB. The TH-MYCN transgenic mouse model and the respective derived HR-NB cell line 9464D closely resemble human NBs in terms of gene expression profiles and tumor phenotype [12]. However, the inefficiency of this cell line to model experimental metastasis has limited the use of this previously designated relevant model for studying HR-NB metastasis in the past. Newly developed HR-NB cell lines with the same C57Bl/6 genetic background address this limitation and provide efficient model systems for experimental metastasis. M1 and M2 cells and M1 variants show high (100%) efficiency in modeling experimental metastasis in immunocompetent mice. Specifically, M1 [19], and M2 consistently develop liver metastasis. Meanwhile M1-2 exhibits bone metastasis by forming tumors in the forelimb, hindlimb, and craniofacial/forehead regions. The exact mechanism underlying the organotropism of these cell lines remains to be elucidated and is a subject of future investigation. Notably, all new cell lines except for M1-2 and M1-6 do not show a significant difference in primary tumor growth relative to the parental 9464D cells, indicative of the specifically acquired metastatic potential of these cell lines.

The in vitro cellular characteristics, such as proliferation, migration, or colony formation, of these cell lines were not higher than those of the 9464D cells. But the specifically acquired anchorage-independent growth phenotype of the newly developed cells may indicate their gained ability to survive the fluid shear flow and proliferate in circulation, eventually colonizing and forming metastases at secondary sites. The anchorage-independent growth ability is reflective of surviving detachment, the initial step of metastasis in vivo [31,32]. Cancer cells that survive the multiple stress conditions upon detachment, such as nutrient deficiency, mechanical force, the loss of growth stimuli, and increased ROS production, can colonize and metastasize at distant organs. Previous studies have shown the association between PI3K-AKT pathway activation and the anchorage-independent survival/growth ability [31], which is consistent with our RNA-seq findings. The gene ontology and pathway analysis of upregulated genes identified the enrichment of PI3K-AKT-pathway-associated genes in M1 cells, providing a possible explanation for the potential of M1 cells to survive in the vasculature and form metastases.

One of the major biological processes upregulated in M1 cells is the increased production of Interlukin-1α (IL-1α). Previous studies have shown the tumor-promoting function of inflammatory cytokine IL-1α in multiple cancer models, such as for liver, lung, ovarian, pancreatic, and head and neck cancers [33,34,35,36,37,38]. Specifically, Watari K. et al. revealed the mechanistic role of IL-1α in promoting angiogenesis and regulating the tumor microenvironment (TME) in the highly metastatic LNM35 lung cancer cells. Moreover, IL-1α expression was significantly associated with distant metastasis and poor patient survival in head and neck squamous cell carcinomas [36]. Cancer-cell-derived IL-1α has previously been shown to induce a tumor-promoting immune microenvironment by activating the cancer-associated fibroblasts in pancreatic cancers [37]. Similarly, IL-1α was reported as an essential inflammatory cytokine promoting colon cancer metastasis by regulating angiogenesis and inducing pro-tumor immune cell infiltration via IL-1α/PI3K/NF-kB signaling. Notably, it was found to be expressed in high levels specifically in the liver’s metastatic colon cancer cell lines, suggesting its correlation with liver metastasis [38]. In line with these previously reported functions of IL-1α in cancer, M1 cells that exhibit increased IL-1α production also demonstrate enrichment in processes such as angiogenesis and blood vessel morphogenesis, positive chemotaxis, and the regulation of macrophage and leukocyte chemotaxis.

The significantly higher invasive ability of M1 cells, along with the observed associated gene expression profile, further explains the aggressive nature of M1 in vivo. Gene ontology analysis identified extracellular matrix (ECM) and extracellular structure organization, tissue remodeling, actin cytoskeleton organization, and cell adhesion processes among the top regulated biological processes in M1 cells. ECM degradation and remodeling is one of the vital events in cancer metastasis, essential for cancer invasion. Importantly, MMP15, MMP28, ADAMTS12, ADAM19, COL4A1, and COL24A1 are some of the upregulated genes associated with these processes.

Matrix metalloproteinases (MMPs) are a family of zinc-dependent endopeptidases that play multidimensional roles at different stages of cancer progression, specifically by remodeling the existing ECM to a tumor-specific ECM. MMPs have been known to promote metastasis by regulating the proteolytic degradation of ECM components, promoting invasion and angiogenesis, regulating the epithelial-to-mesenchymal transition (EMT), and altering cell–cell and cell–ECM interactions [39,40,41,42]. Multivariate analysis identified the association of MMP28 with a poor prognosis in hepatocellular carcinoma. It was found to promote cell migration, invasion, and EMT in hepatic cancer cells [43]. Similarly, another study reported the role of MMP28 in inducing EMT by degrading E-cadherin, thereby promoting metastasis in colon cancer [44]. Likewise, several studies have shown the importance of MMP15 in promoting metastasis in hepatocellular carcinoma and cervical cancer models [45,46].

ADAMS (a disintegrin and metalloproteinase) and ADAMTS (a disintegrin and metalloproteinase with thrombospondin motifs) are a related family of metalloproteinases that, like MMPs, are involved in proteolytic processing, cell adhesion, integrin signaling, and ectodomain shedding. Several studies have indicated their role in cancer progression by regulating the extracellular microenvironment and immune evasion [39,47]. Particularly, ADAM19 has been reported to play pro-tumor or anti-tumor roles, depending on the cancer type. In the context of cancer progression, studies have demonstrated its importance in imparting oncogenic properties to breast cancer cells [48] and its association with poor patient survival in colon cancer [49]. Another study reported the significance of ADAM19 and its proteolytic activity in brain tumors [50]. ADAMTS12 is highly expressed in tumor tissues, and its elevated expression was correlated with a poor prognosis in multiple cancer types, such as pancreatic ductal adenocarcinoma, head and neck cancers, and colorectal and gastric cancers [51,52,53]. Importantly, ADAMTS12 was shown to promote cell invasion and the migration of head and neck cancer cells via the activation of the PI3-AKT pathway [52]. ADAMTS12 increased the transcriptional activation of β-catenin, thereby promoting cell proliferation and migration in colorectal cancer cells [53].

Collagens are the major components of the ECM that are secreted by fibroblasts, cancer cells, and endothelial and immune cells, and their abnormal alignment and high density results in the tumor-specific ECM. This collagen-rich, tumor-specific ECM with increased stiffness is known to promote malignant transformation, intravasation, EMT, and metabolic adaptability and, thus, metastasis [54,55]. Collagens and their abnormal alignment were found to influence the TME by negatively regulating cytotoxic T-cell infiltration and activation in multiple tumor models. Moreover, studies have shown the regulatory role of collagens in driving macrophages towards an M2 phenotype [54]. Specifically, elevated levels of COL4A1 were found in hepatic tumors, and it was shown to promote cell migration and invasion in hepatocellular carcinoma cells via the FAK-Src pathway [56]. Also, the high expression of COL24A1 has been shown to be significantly associated with a poor prognosis in hepatic cancer patients [57].

Thus, the enrichment of these pro-tumor ECM remodelers in the highly invasive M1 cells is potentially responsible for inducing a metastasis-favorable microenvironment at the secondary sites, thereby promoting the colonization of the injected M1 cells. Furthermore, previous studies have shown the oncogenic potential of MMP2/9 in HR-NB cell lines in vitro [58,59,60]; thus, M1 cells provide an opportunity to explore additional, previously unidentified MMPs in HR-NB and their implications in NB-TME in vivo.

In accordance with this gene signature, M1 cells and their derived variants showed resistance towards IFN-γ-induced phenotype changes in vitro, specifically with MHC-I expression, potentially imparting them with the ability to evade immune surveillance in immunocompetent mice. IFN-γ is known to induce an anti-tumor response by enhancing the expression of MHC class I molecules in cancer- and antigen-presenting cells, thereby facilitating the presentation of tumor cells in T-cell-mediated killing [18]. Additionally, studies have reported the tumor-suppressive functions of INF-γ by stimulating blood vessel destruction, apoptosis in cancer cells, the regulation of M1 macrophages, and cytotoxic T-cell infiltration [61,62,63]. An IFN-γ deficiency or insensitivity promoted tumor growth in immunocompetent mice, indicating that IFN-γ is a major regulator of immunosurveillance in immunologically intact hosts [62,64]. This reflects the pathophysiology of some HR-NBs that achieve selective resistance to IFN-γ exposure. M1 and M1-variant cells exhibiting insensitivity to IFN-γ mimic such immune-resistant tumors—importantly, the “immunologically cold” HR-NBs—and, thus, could be suitably exploited to examine immune evading mechanisms in vivo.

Taken together, in vitro characterization and in vivo validation, along with the RNA-seq analysis, reveal the potential mechanisms/pathways that may underly the specifically acquired metastatic ability of the newly developed cell lines. Additionally, we have previously demonstrated extracellular vesicles as one of the mechanisms responsible for the metastatic nature of M1 cells by inducing a pre-metastatic niche at secondary sites [19]. With their high metastatic ability and unchanged MYCN status, M1, M1-variant, and M2 cells reflect HR human NBs. Derived from 9464D cells, which are known to resemble human HR-NB tumors, these new cell lines further provide appropriate systems for studying HR-NB metastasis and examining the TME using C57Bl/6J immunocompetent mice. Moreover, with the ability to feature metastasis in the prominent organs (liver and bone), M1, M2, and M1-2 cells are relevant tools to study HR-NB metastasis and examine the mechanisms underlying organ-specific metastasis in NB. These new cell lines are potentially very promising models for studying metastasis in HR-NBs in an immunocompetent model. In the context of translational research, these newly developed models could be efficiently utilized to test novel therapeutic agents for targeting HR-NB metastasis and examine the potential TME interactions.

## 5. Conclusions

Due to its highly metastatic nature, chemotherapy resistance, and low immunogenicity, HR-NB is among the leading causes of cancer-related deaths in pediatric patients. Despite treatment with aggressive multi-modal radio-chemo-immunotherapy, most patients with metastatic HR-NB develop refractory or relapsed disease, resulting in significantly lower overall survival [65]. Importantly, metastases to different sites exhibit high biological and clinical heterogeneity, thus leading to varied outcomes in patients. It is, thus, essential to better understand the biology of the metastatic process surrounding the microenvironment and cancer cell–immune/stromal cell interactions to develop improved therapeutic strategies to treat metastatic NB. However, to date, there are no suitable immunocompetent in vivo models for studying HR-NB metastasis, showing the characteristic MYCN overexpression. Our study fills this gap by providing newly developed, appropriate, syngeneic HR-NB cell lines that are highly metastatic in nature and feature metastasis in the prominent organs observed in NB patients. Moreover, derived from 9464D tumors that have a similar expression profile and tumor phenotype as those of human HR-NBs, the newly developed cell lines are relevant models for HR-NB metastasis studies. These cell lines are efficient in modeling experimental metastasis, and they can now be utilized by the research community in HR-NB metastasis studies in an immunocompetent system, enabling the examination of the tissue microenvironment and the identification of potential therapeutic targets to treat HR-NB metastasis.

## Data Availability

Data generated in the study are included within the article and its supplementary data files. Additional Metascape raw data files are available upon request from the corresponding author.

## Supplementary Materials

The following supporting information can be downloaded at: https://www.mdpi.com/article/10.3390/cancers15194693/s1. Figure S1: MYCN expression in M1 and M2 cells.; Figure S2: In vitro functional assays.; Figure S3: Quantification of MHC-I expression.; Figure S4: Differentially regulated genes in metastasis-related pathways; Table S1: Differentially regulated genes (RNA-seq data); and Table S2: Enriched GO terms, GO terms for upregulated genes. File S1: Original western blots.
