# Supplementary material for: Generation of Novel Immunocompetent Mouse Cell Lines to Model Experimental Metastasis of High-Risk Neuroblastoma
Source: Cancers (Basel). 2023 Sep 23;15(19):4693. doi: 10.3390/cancers15194693 (PMC10571844; doi:10.3390/cancers15194693)

These western blots correspond to Figure 1B iv).

N-myc expression for all the derived lines (above, gel1).

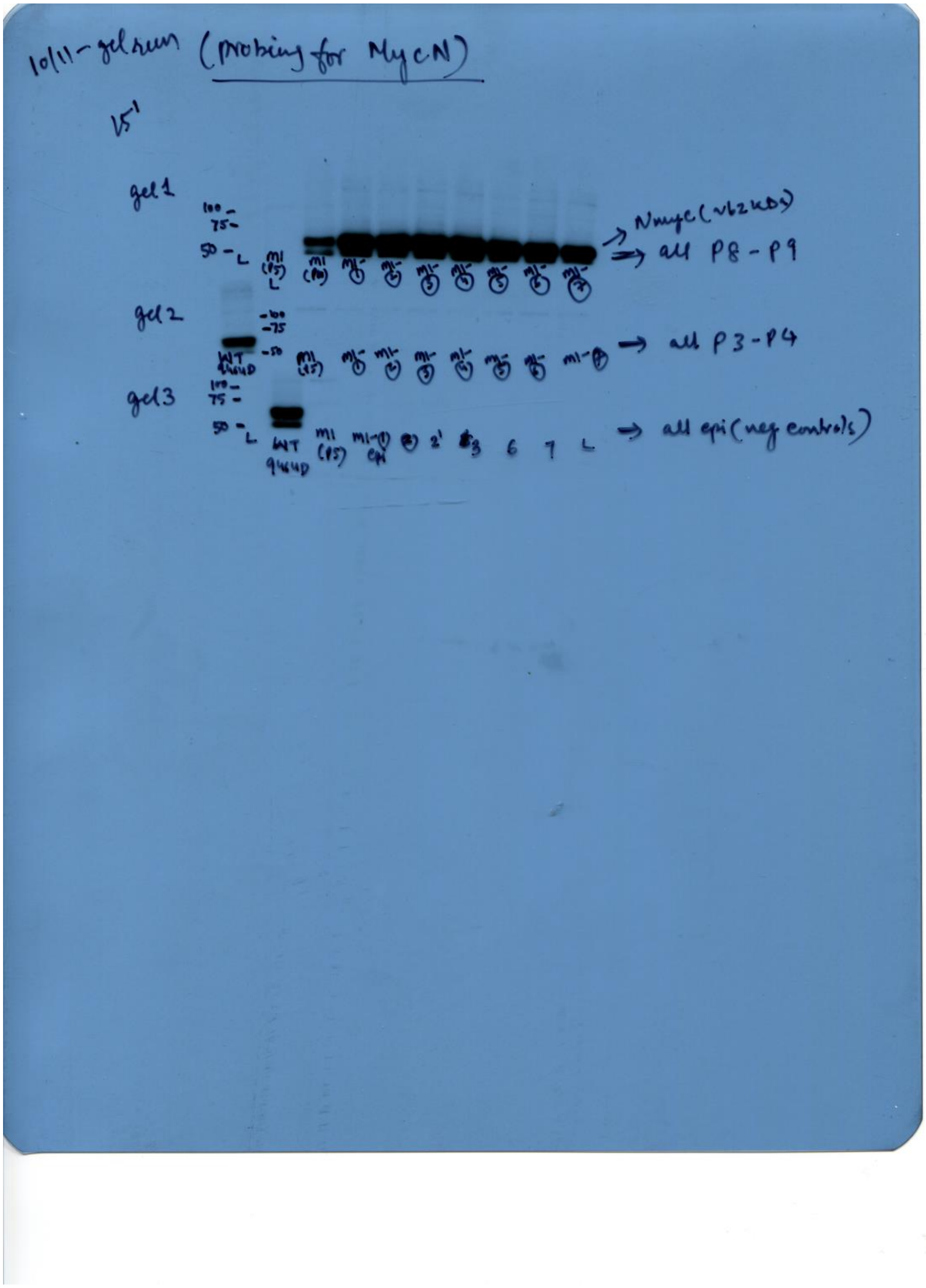

B-actin expression for all the derived lines (above, gel1).

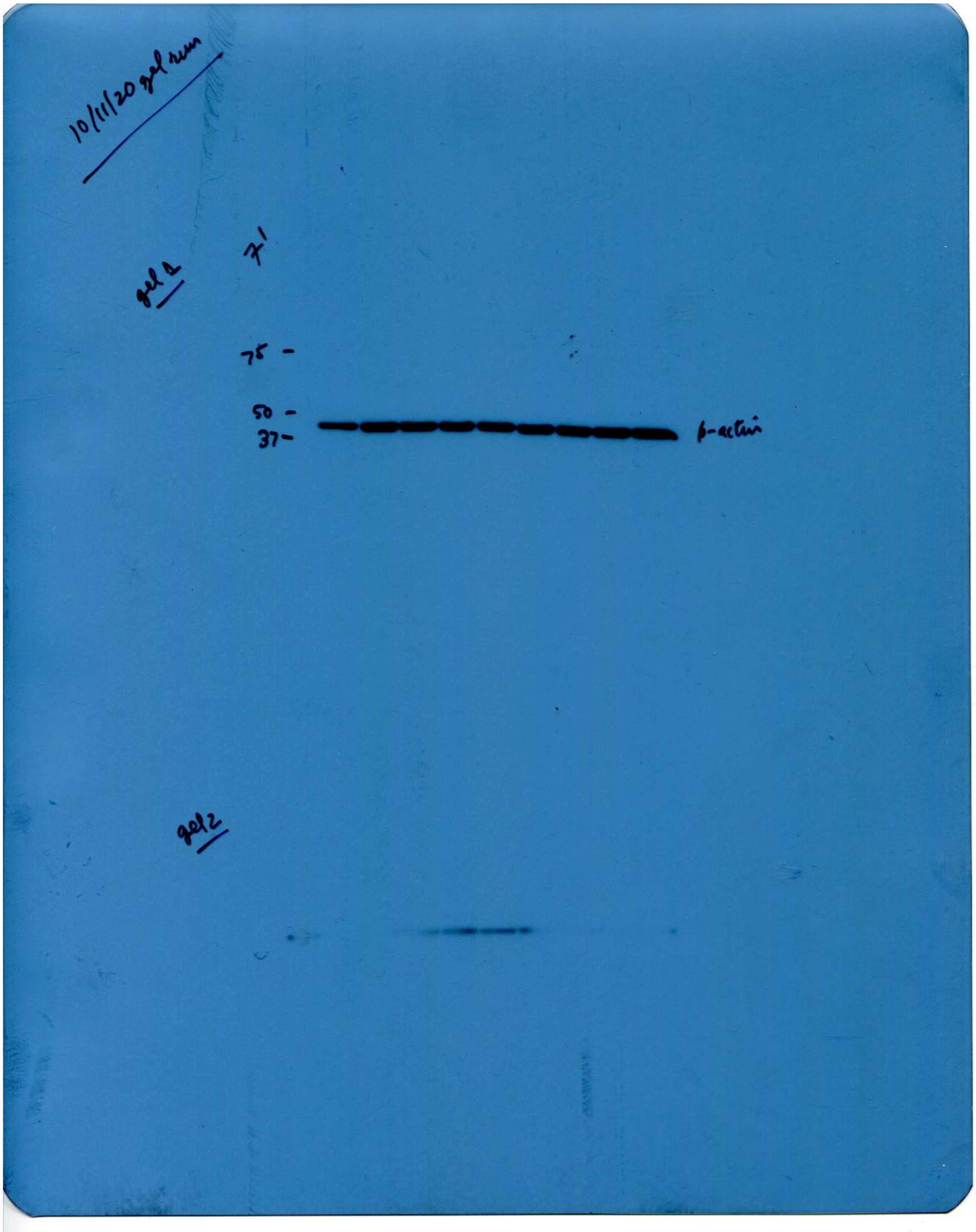

These western blots correspond to Figure 1B iv) for left out non-NB/tissue cells

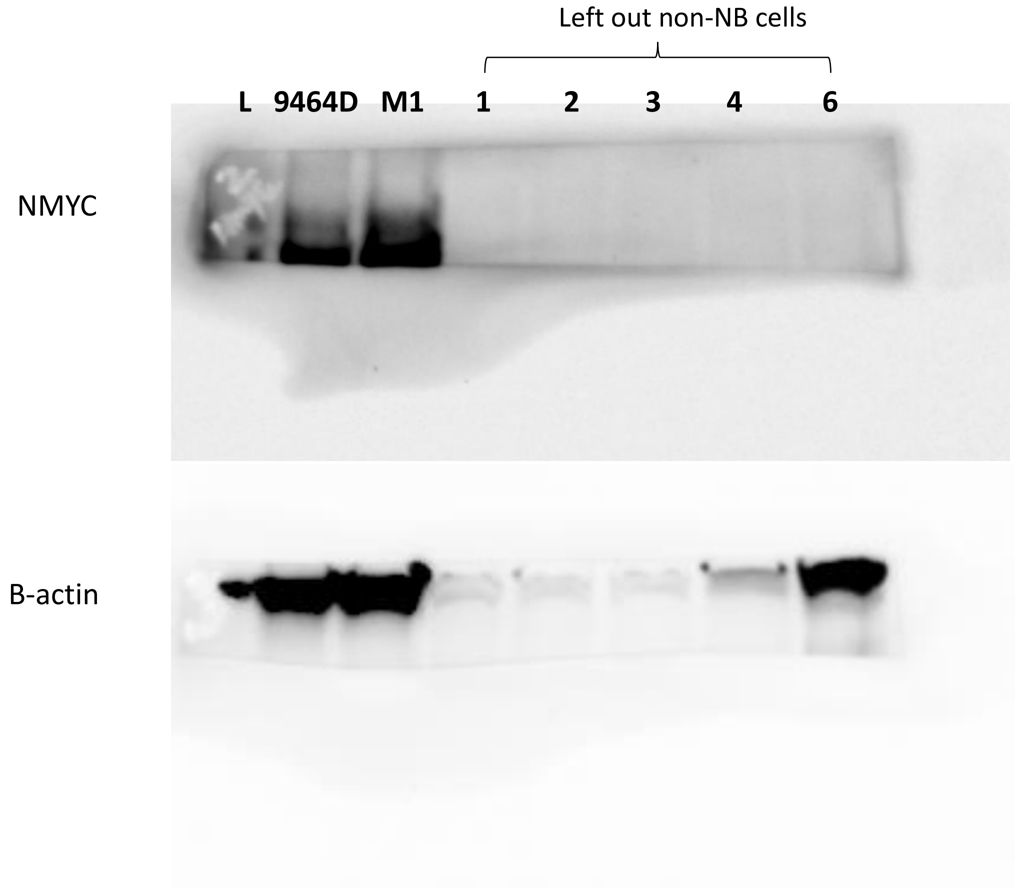

This western blot corresponds to Supplementary Figure S1A.

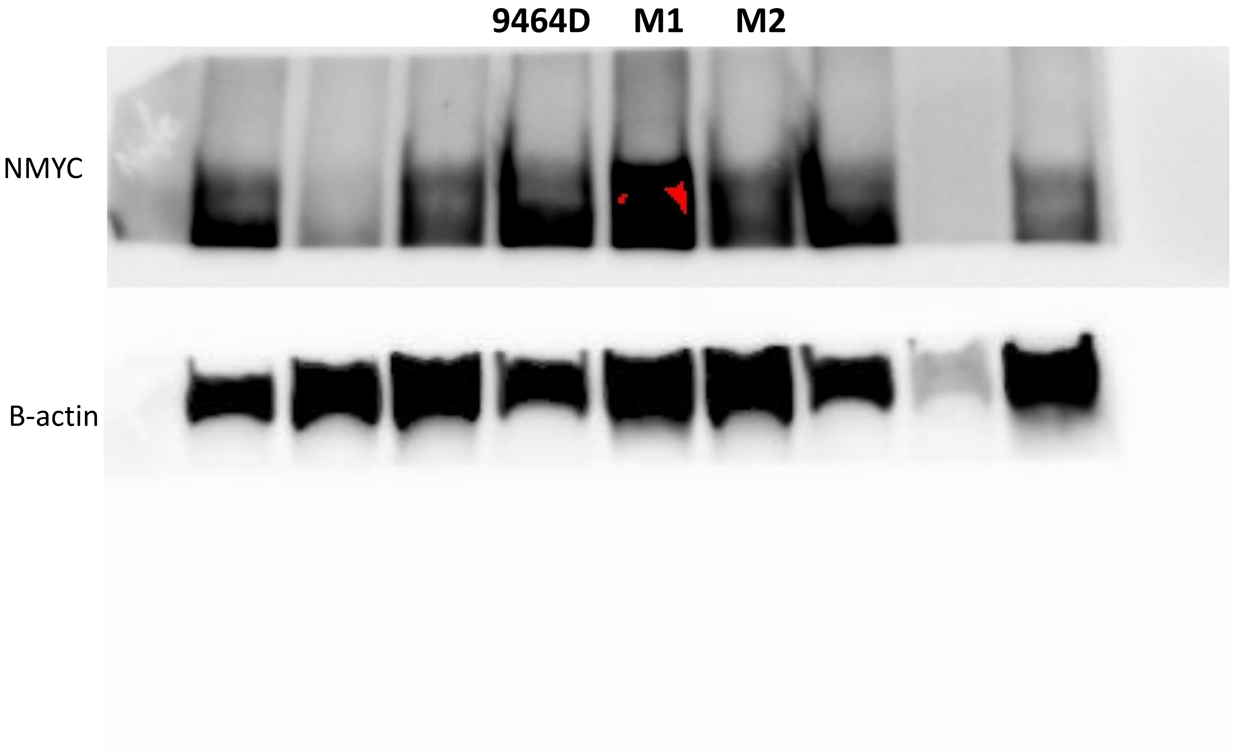

Supplement: Supplementary file 1 [file cancers-15-04693-s001.zip › cancers-2610055-File S1.pdf]
