# Supplementary material for: Generation of Novel Immunocompetent Mouse Cell Lines to Model Experimental Metastasis of High-Risk Neuroblastoma
Source: Cancers (Basel). 2023 Sep 23;15(19):4693. doi: 10.3390/cancers15194693 (PMC10571844; doi:10.3390/cancers15194693)
Supplement: Supplementary file 1 [file cancers-15-04693-s001.zip › cancers-2610055-supplementary.pdf]

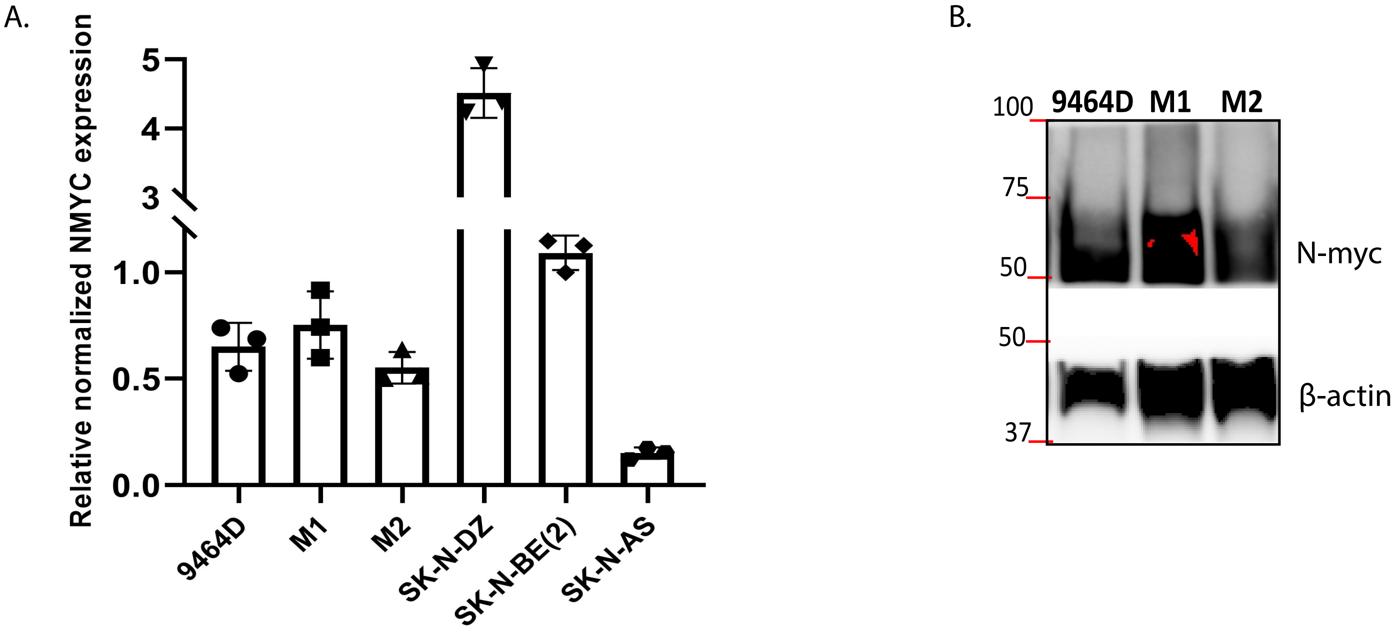

Supplementary figure S1. MYCN expression in M1, M2 cells.

A) RT-PCR indicating (TH-MYCN overexpressed) MYCN transgenic mRNA levels in 9464D, M1 and M2 cells using human MYCN primers (relative normalized expression, normalized to internal reference RPS18, n = 3 independent replicates at different passages) human NB cell lines: SK-N-DZ, SK-N-BE(2) used as positive controls and SK-N-AS used as a negative control for MYCN expression, B) western for total N-Myc protein levels. B-actin used as an internal loading control.

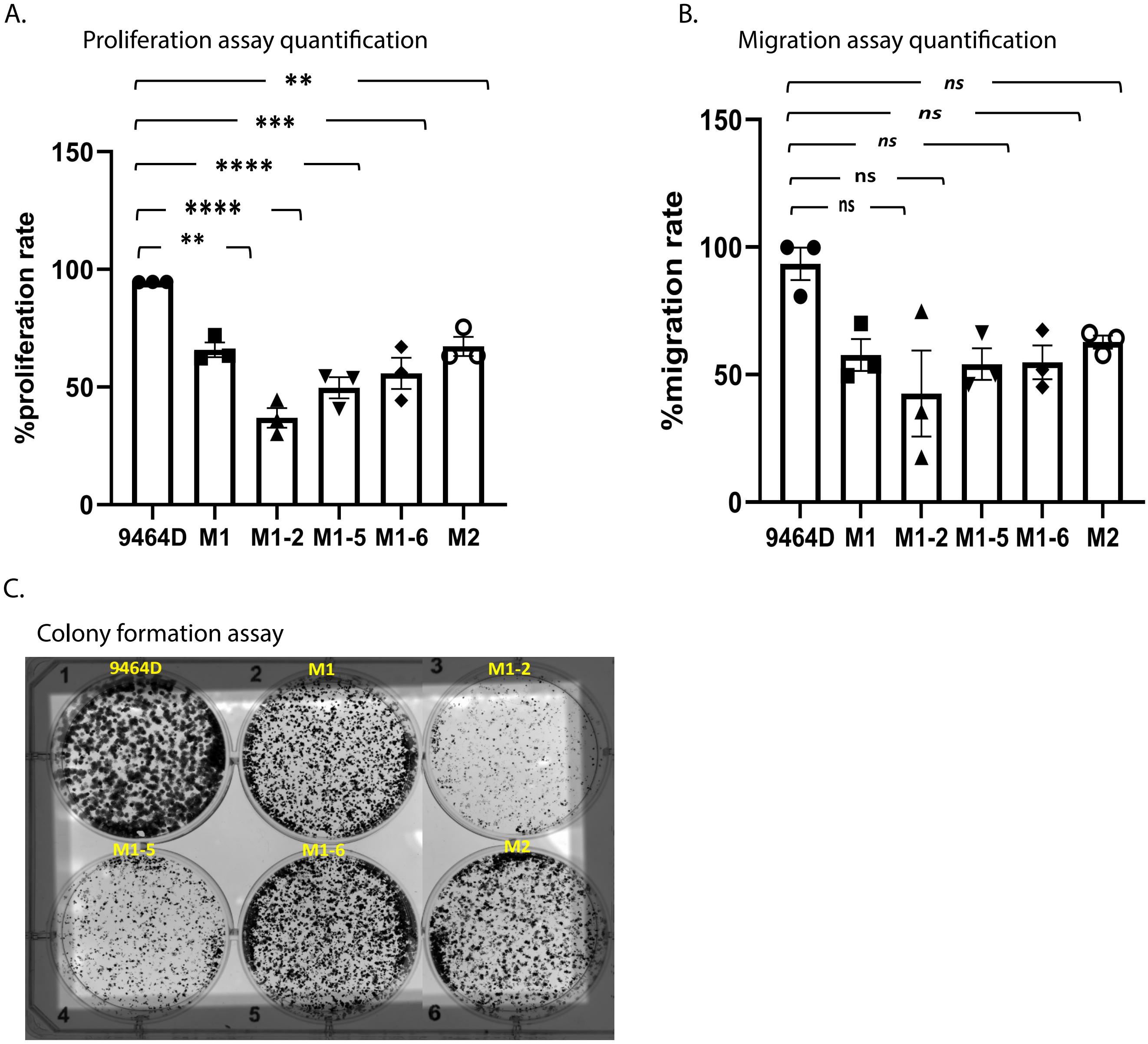

Supplementary figure S2. In vitro functional assays.

A) Cell proliferation assay quantification, statistical analysis – One way ANOVA, mean ± SEM, p-values: 9464D vs M1 – 0.0045, 9464D vs M1-2 < 0.0001, 9464D vs M1-5 < 0.0001, 9464D vs M1-6 - 0.0003, 9464D vs M2 – 0.0067; B) Migration assay quantification, statistical analysis - Brown-Forsythe and Welch ANNOVA, mean ± SEM, p-values: 9464D vs M1 – ns (0.115), 9464D vs M1-2 – ns (0.344), 9464D vs M1-5 – ns (0.083), 9464D vs M1-6 – ns (0.098), 9464D vs M2 – ns (0.122); C) colony formation assay. All quantification data is representative of n=3 experiments.

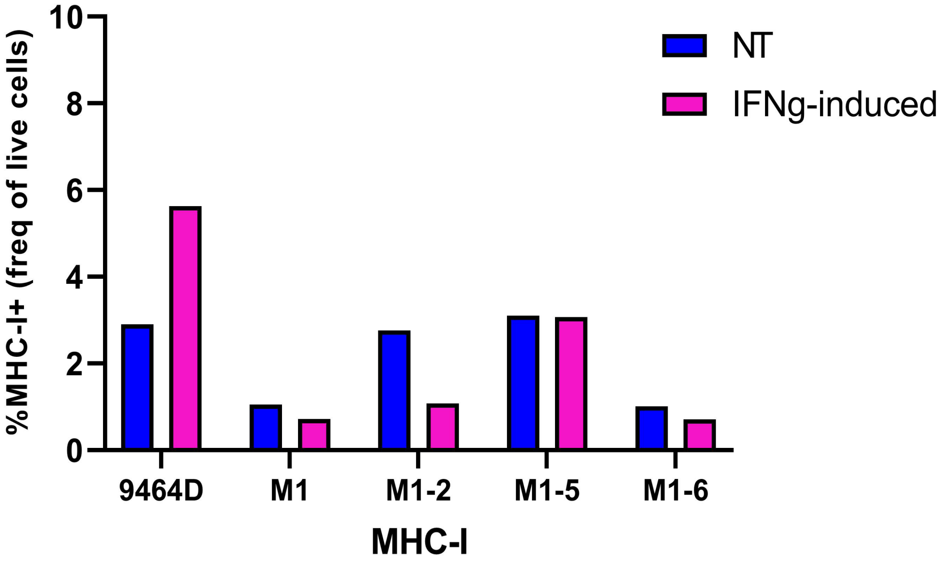

Supplementary figure S3. Quantification of MHC-I expressing 9464D, M1, M1-variant cells. MHC-I expressing cells (% of live cells) upon IFN-γ treatment or no-treatment (NT).

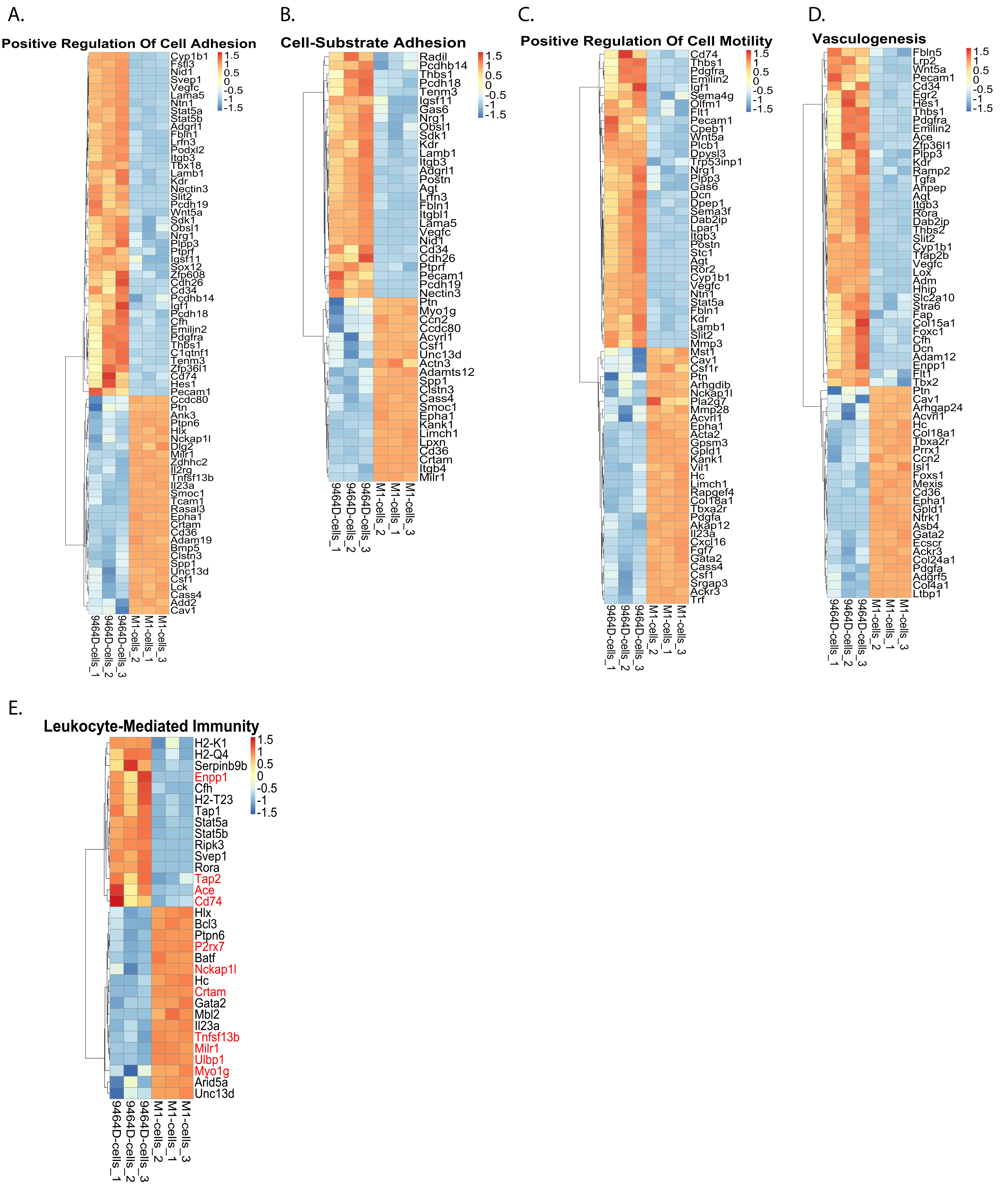

Supplementary figure S4. Differentially regulated genes in metastasis-related pathways.

Heatmaps depicting differentially regulated genes associated with altered biological processes, relevant to metastasis- A) Positive regulation of cell adhesion; B) Cell-substrate adhesion; C) Positive regulation of cell motility; D) Vasculogenesis and E) Leukocyte mediated immunity (surface markers are highlighted in red).
